# Case Report: Kikuchi: The great mimicker

**DOI:** 10.12688/f1000research.14758.1

**Published:** 2018-04-30

**Authors:** Kevin Bryan Lo, Anna Papazoglou, Lorayne Chua, Nellowe Candelario

**Affiliations:** 1 Department of Medicine, Einstein Medical Center, Philadelphia, PA, 19141, USA

**Keywords:** lymphadenitis, fever, Kikuchi, autoimmune

## Abstract

Kikuchi-Fujimoto disease is a form of a benign necrotizing lymphadenitis which is most commonly misdiagnosed as tuberculosis and or lymphoma, usually more common among young adults in Asia. It is a benign disease but can mimic a lot of other disease processes spanning infectious, rheumatologic and even hematologic malignancies. Our patient presented with prolonged fever and lymphadenopathy. Initial considerations were lymphoma and a nonspecific viral infection. A CT scan showed diffuse cervical lymphadenopathy with lacrimal gland involvement. An excisional lymph node biopsy was done which revealed Kikuchi disease. Patient was given steroids with immediate response with defervescence. Kikuchi is a disease with many mimics and a complete workup is needed to exclude serious disease like malignancy.

## Introduction

Kikuchi-Fujimoto disease (sometimes also known as Kikuchi Disease) is usually more common among young adults in Asia. It is a benign disease, but can mimic a lot of other disease processes spanning infectious, rheumatologic and even hematologic malignancies ^1^. It usually presents with fever and cervical lymphadenopathy but occasionally it can manifest together with other unusual symptoms further increasing the chances of misdiagnosis.

## Case

A 20-year-old African American woman with no other known prior medical history, presented to our institution January 2018 with fevers of 3 weeks’ duration. The fevers were predominantly in the late afternoon hours, associated with night sweats, frontal headache, tender cervical lymphadenopathy, anorexia and malaise.

Two weeks prior she saw her primary care physician who diagnosed her with viral illness and recommended supportive care. She also visited the emergency department and was diagnosed with lymphadenitis; a course of amoxicillin/clavulanic acid was prescribed of unrecalled dose and she wasn’t able to finish the whole course. Symptoms however persisted, and the patient also developed bilateral periorbital swelling and non-bloody diarrhea prompting her presentation at our institution. The patient indicated they had no cough, chest pain, dysuria, abdominal pain, arthralgia, rash, recent travel or sick contacts.

The patient was not in distress, with blood pressure of 120/70 mm Hg, febrile to 39.6 C and tachycardic with heart rate of 110 bpm. Physical exam was notable for bilateral periorbital swelling with violet discoloration of the eyelids, conjunctival pallor and painless bilateral cervical lymphadenopathy. No rash or joint swelling was noted.

Complete blood count revealed leukopenia with a white cell count of 2.9 × 10 ^3 ^/mcL (65% neutrophils, 13% lymphocytes, 13% bands), microcytic anemia with a hemoglobin of 8.5 gr/dL (mean corpuscular volume 65 fL) and 181 × 10 ^3 ^/mcL platelets. C-reactive protein (CPR) and erythrocyte sedimentation rate (ESR) level were markedly elevated at 51 and 84 respectively. Lactate dehydrogenase (LDH), ferritin and haptoglobin were also elevated. The patient tested negative for β-human chorionic gonadotropin (hCG), HIV, hepatitis B and C, angiotensin converting enzyme (ACE), antinuclear antibodies (ANA) and rheumatoid factor (RF).

**Figure 1.**
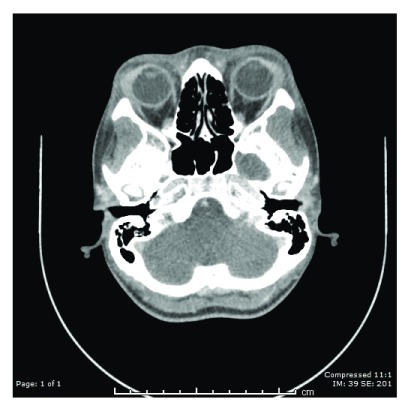
Computed tomography (CT) scan coronal view head with contrast. Bilateral lacrimal glands appear large with mild increased enhancement.

**Figure 2.**
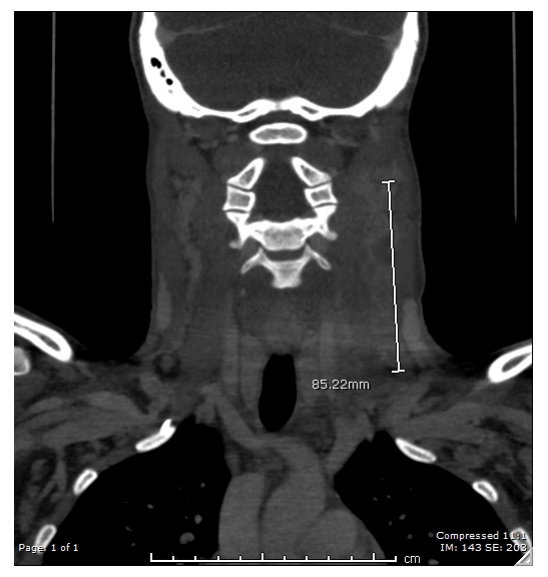
Computed tomography (CT) scan sagittal view neck with contrast. Bilateral left greater than right cervical jugular chain, level I, occipital and supraclavicular lymph nodes demonstrate heterogeneous enhancement and enlargement, largest demonstrating conglomeration and cystic changes along the left jugular chain measuring up to 8.5 cm in length of the conglomerate.

Computed Tomography of the neck revealed bilateral cervical lymphadenopathy, enhancement and mild enlargement of the parotid and lacrimal glands and diffuse swelling of the pharyngeal mucosa and marked enhancement of bilateral cervical soft tissue planes [ Figure 1 and Figure 2].

**Figure 3.**
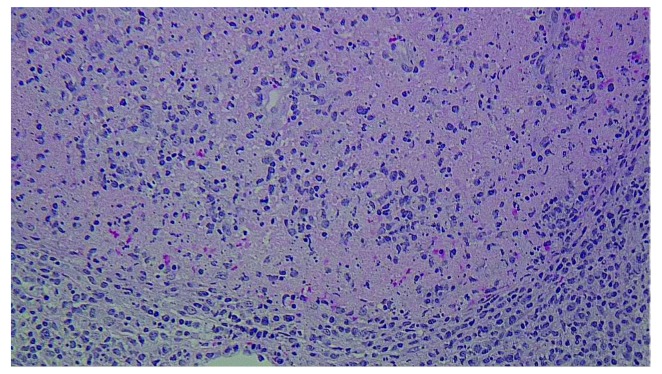
Histopathology of cervical lymph node. Geographical necrosis with fibrinoid deposits and nuclear fragments with apoptotic cells. Surrounding this area are pale histiocytes and lymphocytes. Neutrophils and eosinophils are characteristically absent.

She was observed off antibiotics. Blood cultures, serology for Epstein Barr virus (EBV) and cytomegalovirus (CMV), bone marrow biopsy and flow cytometry were all negative. Excisional biopsy of the left cervical lymph node revealed characteristic findings of Kikuchi-Fujimoto disease which showed geographic necrosis with fibrinoid deposits and apoptotic cells surrounded by a mononuclear infiltrate characteristically without neutrophils and eosinophils [ Figure 3]. The patient was started on prednisone 40mg per day with rapid resolution of symptoms. Steroids were tapered after one week of treatment. Upon follow up in Rheumatology clinic 4 months later, patient was noted to be completely symptom free.

## Discussion

Kikuchi disease was first independently described through case series in 1972 by Kikuchi and Fujimoto as a form of a benign necrotizing lymphadenitis which was most commonly misdiagnosed as tuberculosis and or lymphoma ^2^. The main etiology for Kikuchi disease is still unknown but there are various studies that implicate viruses such as EBV as a potential trigger ^2, 3^. It is also closely related to systemic lupus erythematosus (SLE) and in fact, there are studies and case reports showing a strong association between the two disease processes with the diagnosis of SLE coming before, after or even simultaneously with Kikuchi disease ^4, 5^. The most frequent presenting symptom was fever while the most common presenting sign was cervical lymphadenopathy ^5^. It also presents together with constitutional symptoms like night sweats and weight loss which can be initially be misdiagnosed as tuberculosis or lymphoma ^1, 2^. However, Kikuchi disease has also been implicated to cause a wide range of symptoms ranging from neurological, musculoskeletal, cutaneous and glandular dysfunction ^6^. Eye manifestations for Kikuchi usually present as uveitis and conjunctivitis ^6, 7^. Our case is unique because bilateral eyelid swelling has only been reported twice in the literature as a possible presentation of Kikuchi disease, this may be attributed to lacrimal gland involvement which was seen in the imaging findings in our patient ^6, 8, 9^. Definitive diagnosis is established by lymph node biopsy. Classic biopsy findings include necrosis without a neutrophilic infiltrate with the predominance of histiocytes and T lymphocytes ^1, 2^. Kikuchi is a benign self-limiting disease and symptoms usually resolves spontaneously within 4 months in majority of cases with supportive treatment ^1, 6^. The use of glucocorticoids have been found to have some benefit but is usually reserved in more severe persistent cases ^5, 6, 10^. Kikuchi is a disease with a lot of mimics, the amount of workup alone together with the actual disease manifestations can lead to a lot of morbidity and discomfort for the patient. Nevertheless, a complete workup including an excisional biopsy is recommended to help rule out other serious diseases like malignancy. Close follow up is also needed to monitor for the development of closely associated rheumatological diseases like SLE. Strengths in the approach of the case was the exhaustive diagnostic approach used to arrive at the correct diagnosis for the patient. All possible differentials were considered especially the serious ones such as malignancy. Weakness involved were due to the extensive workup done which consisted of numerous blood tests and invasive tests such as a biopsy, this caused a significant degree of anxiety and morbidity to the patient as well.

## Conclusions

Kikuchi is a great mimicker and can be confused with tuberculosis, lymphoma and other viral illnesses. A complete workup including an excisional biopsy is recommended to help rule out other serious diseases like malignancy. Close follow up is needed to monitor for the development of closely associated rheumatological diseases like SLE.

## Ethics and consent

Written informed consent for publication of their clinical details and clinical images was obtained from the patient and parent.

## Data availability

All data underlying the results are available as part of the article and no additional source data are required.
