# Supplementary material for: Case Report: Kikuchi: The great mimicker
Source: F1000Res. 2018 Apr 30;7:520. [Version 1] doi: 10.12688/f1000research.14758.1 (PMC6081987; doi:10.12688/f1000research.14758.1)
Supplement: Peer review history

# Peer review: Referee response for version 1

**DOI:** 10.5256/f1000research.16056.r35937

**Published:** 2018-08-07

**Authors:** Arvind P. Ganpule, Tak Gopal Ramdas

**Affiliations:** 1 Department of Urology, Muljibhai Patel Urological Hospital (MPUH), Nadiad, India; 2 Muljibhai Patel Urological Hospital (MPUH), Nadiad, India

Explanation for second referee question:

 Periodic acid-Schiff (PAS) stain , Ziehl Neelsen (ZN) stain, Genexpert examination for tubercle bacilli (TB), immmunohistochemistry (IHC) examination for lymphoma, tests for systemic lupus erythematosus (SLE) to rule out different differentials, were not mentioned.

 Key features on lymph node biopsy are fragmentation, necrosis and karyorrhexis were not mentioned by authors in the histopathology examination.

 Treatment includes symptomatic care, analgesics-antipyretics, corticosteroids and not the corticosteroid alone as mentioned by authors.

 Author may put “Ganpule AP, Chabra JS, Singh AG, Tak GR, Soni S, Sabnis R, Desai M. Case Report: Kikuchi-Fujimoto disease: a diagnostic and therapeutic dilemma following pretransplant nephrectomy for a 2.35 Kg kidney. F1000Research. 2016;5” as the reference for their article.

We have read this submission. We believe that we have an appropriate level of expertise to confirm that it is of an acceptable scientific standard.

# Peer review: Referee response for version 1

**DOI:** 10.5256/f1000research.16056.r35130

**Published:** 2018-06-25

**Authors:** Abdou-Rajack Ndiaye, Cheikh Lame

**Affiliations:** 1 Service de Médecine interne, Hôpital Principal de Dakar, Dakar, Senegal; 2 Service d'Oto-Rhino-Laryngologie, Hôpital Principal de Dakar, Dakar, Senegal; 3 Hôpital d'Instruction des Armées Sénégalaise, Dakar, Senegal

The article is correctly written. It is an original one. The described pathology is uncommon and raises diagnostic problems. References are up-to-date.

 However, some minor revisions must be done:

1. Serodiagnostic tests for toxoplasmosis, rubella, syphlis, and infectious mononucleosis must be performed in presence of chronic lymphadenopathy with fever, even they are often negative in such case.
2. Precise the place of immunohistochemical study (cd68, cd8) to confirm the diagnosis.
3. Deaths are rare but have been observed in systemic forms of kikuchi disease.
4. Recent treatment with intravenous immunoglobulins has been proposed in severe and resistant forms.
5. Figure 3: Specify the used coloration and the corresponding magnification.

We have read this submission. We believe that we have an appropriate level of expertise to confirm that it is of an acceptable scientific standard.

## References

[1] LameCALoumBFallAKCucheroussetJNdiayeAR: Kikuchi-Fujimoto disease, a rare cause of lymphadenopathy in Africa. Description of the first case in Senegal and review of the literature. *Eur Ann Otorhinolaryngol Head Neck Dis*.2017;134(5) : 10.1016/j.anorl.2017.02.007 347-349 10.1016/j.anorl.2017.02.007 28279633
